# Association Between Interventional Cardiologist Practice Characteristics, Coronary Artery Bypass Grafting Use, and Clinical Outcomes

**DOI:** 10.1093/ejcts/ezag188

**Published:** 2026-06-23

**Authors:** Justin Blackman, Stephanie Quon, Darrel P Francis, James P Howard, Aaryan Dwivedi, Kevin Makasoff, Yousif Ahmad, Kanwal Kumar, Laura Arbour, Andrew D Krahn, Markus B Sikkel

**Affiliations:** Island Medical Program, Faculty of Medicine, University of British Columbia, Victoria, BC V8P 5C2, Canada; Vancouver Fraser Medical Program, Faculty of Medicine, University of British Columbia, Vancouver, BC V6T 1Z3, Canada; National Heart and Lung Institute, Imperial College, London SW7 2AZ, United Kingdom; National Heart and Lung Institute, Imperial College, London SW7 2AZ, United Kingdom; Vancouver Fraser Medical Program, Faculty of Medicine, University of British Columbia, Vancouver, BC V6T 1Z3, Canada; Southern Medical Program, Faculty of Medicine, University of British Columbia, Kelowna, BC V1V 1V7, Canada; Division of Cardiology, University of California San Francisco, San Francisco, CA 94143, United States; Royal Jubilee Hospital, Division of Cardiac Surgery, University of British Columbia, Victoria, BC V8R 1J8, Canada; BC Children’s Hospital, Department of Medical Genetics, University of British Columbia, Vancouver, BC V6H 3N1, Canada; St. Paul’s Hospital, Division of Cardiology, University of British Columbia, Vancouver, BC V6Z 1Y6, Canada; Royal Jubilee Hospital, Division of Cardiology, University of British Columbia, Victoria, BC V8R 1J8, Canada

**Keywords:** coronary artery bypass grafting, percutaneous coronary intervention, interventional cardiology, revascularization, referral, practice variation, cardiology

## Abstract

**Objectives:**

Quantify inter-operator variation in referred coronary artery bypass grafting (CABG) following angiography and evaluate associations with practice patterns and long-term outcomes.

**Methods:**

Observational study using administrative health data in British Columbia, Canada (2010-2024). Interventional cardiologist-level *CABG Rate* was defined as the proportion of referred CABGs to total angiograms performed. Variation across Interventionalists was correlated with other practice characteristics. Among patients undergoing angiography followed by revascularization, associations between operator *CABG Rate* and all-cause mortality, major adverse cardiovascular events (MACEs), and repeat revascularization were evaluated using hierarchical Cox regression.

**Results:**

Among 252 408 angiograms by 40 Interventionalists, *CABG Rate* varied 13-fold (2.03%-26.4%) and was not explained by hospital-level factors alone (intraclass correlation coefficient [ICC]: 0.358; 95% confidence interval [CI], 0.054-0.621). Higher *CABG Rates* were associated with lower percutaneous coronary intervention (PCI) utilization (R = −0.60; *P* < .001), lower PCI extensiveness (R = −0.52; *P* < .001), and lower procedural volume (R = −0.36; *P* = .022). In 73,603 first-time revascularized patients, *CABG Rate* was associated with reduced repeat revascularization (hazard ratio [HR] = 0.075; *P* < .001), without differences in mortality or MACE.

**Conclusions:**

Referred CABG varies markedly between Interventionalists and reflects operator practice style. Higher CABG utilization is associated with more durable revascularization without impact on survival or MACE. Broader implementation of multidisciplinary Heart Teams may improve consistency of care.

## Graphical abstract

**Figure:**
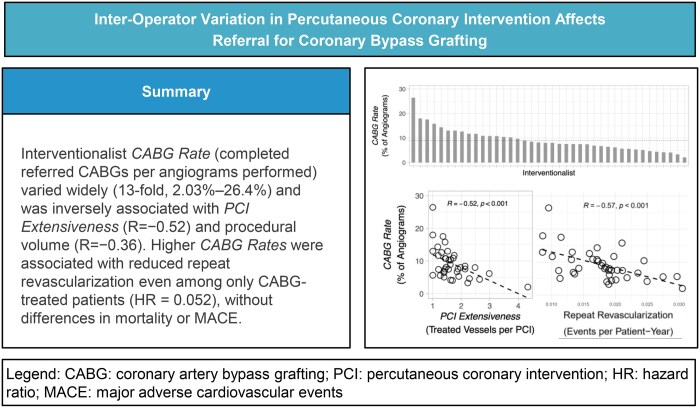

## Introduction

Coronary artery disease is frequently treated via revascularization over and above optimal medical therapy. Approximately 182 per 100 000 patients undergo percutaneous coronary intervention (PCI) and 49 undergo coronary artery bypass grafting (CABG) in the United States annually^1^; in comparison, annual rates are approximately 155 PCI^2^ and 43 CABG^3^ in Canada. The choice between procedures depending on clinical context is contentious globally.^4^^,^^5^ For example, randomized trials report conflicting superiority of revascularization strategy for left main or multivessel coronary artery disease.^6^

What remains largely unexamined is the extent to which this decision varies with the physician who plays a vital role in determining whether to proceed with PCI, refer for surgery, or initiate Heart Team discussion—namely, the interventional cardiologist (Interventionalist) performing the initial angiography.

We examined variations in CABG following angiography across treating Interventionalists in British Columbia, Canada (BC) from 2010 to 2024. We introduce a novel metric, the *CABG Rate*, to quantify the likelihood that an average patient of an Interventionalist undergoing an angiogram will subsequently undergo CABG. We subsequently assess how this rate relates to other practice measures such as their PCI utilization, procedural extensiveness, and procedural volume. We aimed to evaluate the association between a provider’s CABG Rate and long-term patient outcomes.

## Methods

### Data source and study population

This observational study was approved by the Institutional Review Boards of Vancouver Island Health Authority and The University of British Columbia (H24-01169), who waived requirements of informed consent from patients due to the de-identified and retrospective nature.

We obtained records using the BC Health Data Platform. We accessed the Medical Services Plan billing records^7^ of the provincial universal single payer public health plan serving 5.5 million persons which provided information on every CABG, angiogram, and PCI performed in adults aged 20 to 79 (see **eAppendix S1** in **Supplement S1**). In BC, diagnostic angiography and PCI procedures are performed in centralized catheterization laboratories that each serve a defined regional catchment area. Within each centre, Interventionalists typically treat patients drawn from the same regional referral population, with case allocation determined primarily by procedural scheduling or on-call availability rather than geographic district. We restricted our analysis to those patients receiving angiograms by Interventionalists who performed at least 375 angiograms over the study period, in order to limit extreme findings from small sample sizes.

We accessed the Ministry of Health Chronic Disease Registry,^8^ providing the dates of every myocardial infarction (MI), diabetes, kidney disease, heart failure, lung disease, and stroke diagnosis occurring within BC. We accessed the Vital Statistics Agency Vital Events records,^9^ with every registration of death in the province. Comorbidities and prior procedural history were identified using the full available historical records in the datasets, which extend back to at least 1992; the study start date was selected to reflect contemporary practice while allowing an extended look-back period to identify relevant prior events. The data were extracted on April 25, 2025.

### Definitions

The key outcome measure that we defined for each Interventionalist was *CABG Rate.* This was the number of completed CABG procedures referred by an individual Interventionalist, divided by the number of angiograms performed by that same Interventionalist over the same period. We further quantified other aspects of the Interventionalists’ practice to assess correlation with the *CABG Rate*. The *PCI Rate* was the number of PCI cases an Interventionalist performed divided by the number of angiograms they performed over the same time period. *PCI Extensiveness* was defined as the average number of coronary vessels treated with PCI billed across an Interventionalist’s PCI cases (see **eAppendix S2** in **Supplement S1**). *CABG Extensiveness* was the average number of bypass grafts performed per referred CABG case. *Cases Per Day* measured the average number of angiogram cases an Interventionalist billed per day in the catheterization lab: because PCI procedures are almost always performed in conjunction with an angiogram (95.4% of PCI cases in this dataset), this metric primarily reflected operator procedural volume. *Referred PCI Rate* was calculated as the number of referred PCI procedures performed by a separate Interventionalist divided by the number of angiograms performed by the referring Interventionalist. The *Non-Invasive Rate* was calculated as 1 minus the *CABG Rate*, the *PCI Rate*, and the *Referred PCI Rate*, reflecting the proportion of angiogram patients that received no subsequent invasive intervention (medical therapy only).

### Statistical analysis

Practice pattern associations were assessed using correlation (R). Temporal variations were summarized using linear regression to illustrate overall linear trends. To quantify differences in practices between practitioners and across hospitals, we used linear mixed-effects regression with hospital included as a random intercept, allowing the model to account for clustering of Interventionalists within hospitals recognizing that operators practicing at the same site share local institutional factors: we quantified variations using the intraclass correlation coefficient (ICC) and evaluated for significance using the Kruskal-Wallis test. A significance threshold of 0.05 was used for all statistical tests. R version 4.4.0 was used for all analysis.^10^

### Secondary analysis to reduce case mix confounding

We identified potential for confounding whereby cases more or less suitable to CABG might be assigned to Interventionalists with particular practice styles or skill sets. For example, patients with extensive prior revascularization may be directed to operators with particular procedural expertise, which could lead to systematic bias in case mix. To mitigate this, we conducted a secondary analysis restricted to patients experiencing acute MI who had no prior history of MI, angiogram, CABG, or PCI (see **eAppendix S3** in **Supplement S1**).

### Clinical outcomes in revascularized patients

In a third cohort, we identified all patients with no prior coronary angiography or revascularization who underwent index angiography by providers included in the primary analysis and subsequently underwent CABG or PCI within 60 days. Time-to-event analyses were performed to evaluate the association between a provider’s *CABG Rate* and outcomes of patients in this cohort, including overall survival, 3-point major adverse cardiovascular events (MACEs; composite of nonfatal MI, nonfatal stroke, or cardiovascular death^11^), and repeat-revascularization-free survival. Unadjusted Kaplan-Meier curves were constructed with patients stratified by the tertile of the angiography provider’s *CABG Rate*.

Cox proportional hazards models were fitted to estimate the association between *CABG Rate* and patient outcomes after adjustment for differences in case mix. Covariates included provider *CABG Rate*, patient age group, and angiogram date modelled using restricted cubic splines, whether treatment was for acute MI, and comorbidities including prior MI, diabetes, hypertension, chronic obstructive pulmonary disease, heart failure, chronic kidney disease, and prior stroke or transient ischaemic attack. Robust variance estimates were used to account for clustering at the hospital level.

## Results

In total, 54 Interventionalists that billed for at least 1 angiogram during the study period were identified; 14 were excluded for performing fewer than 375 angiograms over the study period (range 1-42 procedures), leaving 40 Interventionalists included in the analysis. As a result, 118 angiograms were excluded from the study (0.05% of all records). In total, 252 408 angiograms, 104 020 PCI procedures, and 20 203 CABG operations, performed between 2010 and 2024 in BC, were included. These involved 198 275 patients and 48 cardiac surgeons.

### CABG rates and practice patterns

**Figure 1.**
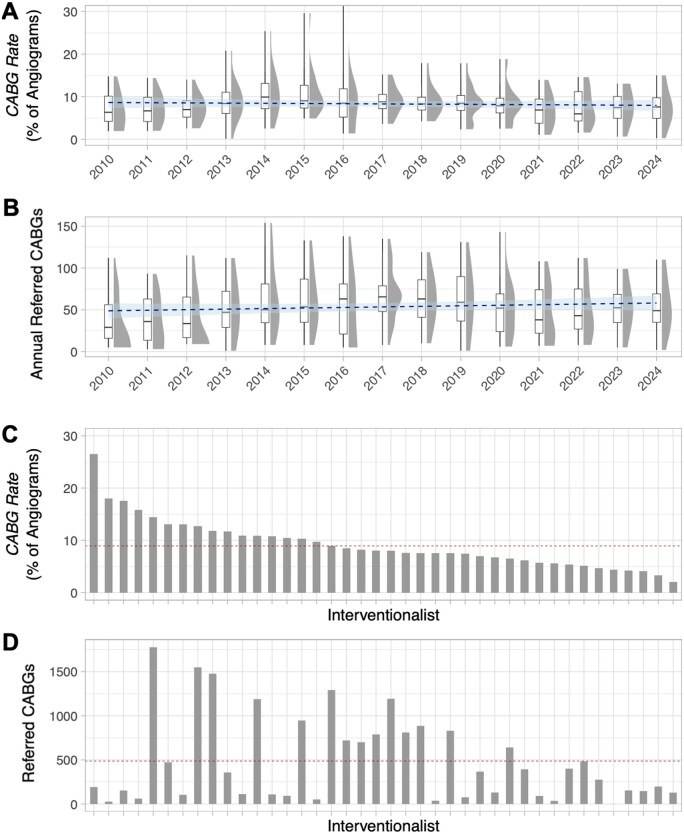
*CABG Rate* by Year and Interventionalist. (A) Distribution of *CABG Rate* across interventionalists by year. The dashed trend line represents the mean linear temporal trend, and the shaded band represents its 95% confidence interval. (B) Distribution of total referred CABG procedures across interventionalists by year. (C) *CABG Rate* by interventionalist across all study years. The horizontal dashed reference line indicates the overall mean across interventionalists. (D) Total referred CABG procedures by interventionalist across the study period, presented in the same ordering as panel C. Abbreviation: CABG, coronary artery bypass grafting.

The mean *CABG Rate* across all years and Interventionalists was 8.53%. Annual *CABG Rates* across all Interventionalists remained stable over time, with a linear trend of only −0.04%/year (*P* = .43) (**Figure 1A**). Total CABG cases by referring Interventionalist are shown in **Figure 1B**, with no significant linear temporal trend (*P* = .21). However, *CABG Rate* demonstrated considerable variation across Interventionalists from 2.03% to 26.4% (**Figure 1C**); there was a 13-fold variation in the likelihood of a patient undergoing CABG depending on the Interventionalist performing the angiogram. The distribution of total referred CABG procedures by Interventionalist is shown in **Figure 1D**.

**Figure 2.**
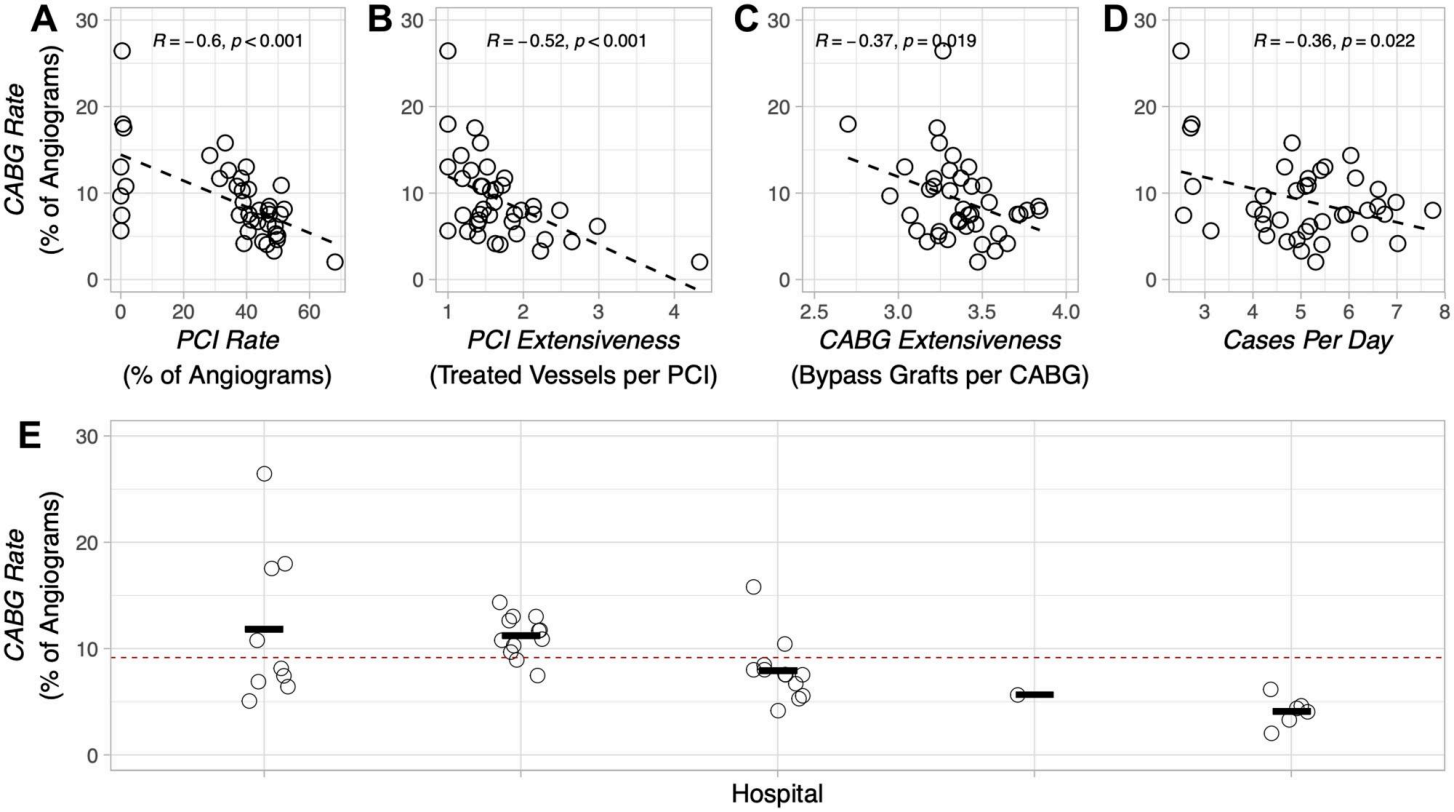
*CABG Rate* by Interventionalist Factor and Hospital. Each circle represents one interventionalist. (A) *CABG Rate* versus *PCI Rate*. (B) *CABG Rate* versus PCI Extensiveness. (C) *CABG Rate* versus *CABG Extensiveness*. (D) *CABG Rate* versus *Cases Per Day*. (E) *CABG Rate* by hospital. Horizontal summary bars indicate the average CABG Rate for each hospital, and the horizontal dashed reference line indicates the overall study mean. Abbreviations: CABG, coronary artery bypass grafting; PCI, percutaneous coronary intervention.

The mean *PCI Rate* across all years and all Interventionalists was 35.0%, with individual *PCI Rates* varying from 0.00% to 68.1%. *CABG Rate* and *PCI Rate* were negatively correlated (R = −0.60, *P* < .001; **Figure 2A**). Patients who underwent angiography by Interventionalists who treated more vessels per PCI case were less likely to undergo CABG (R = −0.52, *P* < .001; **Figure 2B**). Patients of Interventionalists that referred for CABG more frequently tended to have fewer bypass grafts performed in CABG (R = −0.37, *P* = .02; **Figure 2C**). Procedural volume was negatively associated with referred CABG (R = −0.36, *P* = .02; **Figure 2D**): patients of high-volume catheterization lab operators received surgical revascularization less frequently.

Utilization of CABG varied significantly across the 5 included hospitals. Hospital averaged *CABG Rates* across Interventionalists ranged from 4.09% to 11.9% (*P* < .001) (**Figure 2E**). These patterns exhibited an ICC of 0.358 (95% confidence interval [CI], 0.054-0.621), meaning only 35.8% of the variance in *CABG Rate* observed across operators was attributable to differences between hospitals, and the remainder occurred independent of hospital-level effects.

**Figure 3.**
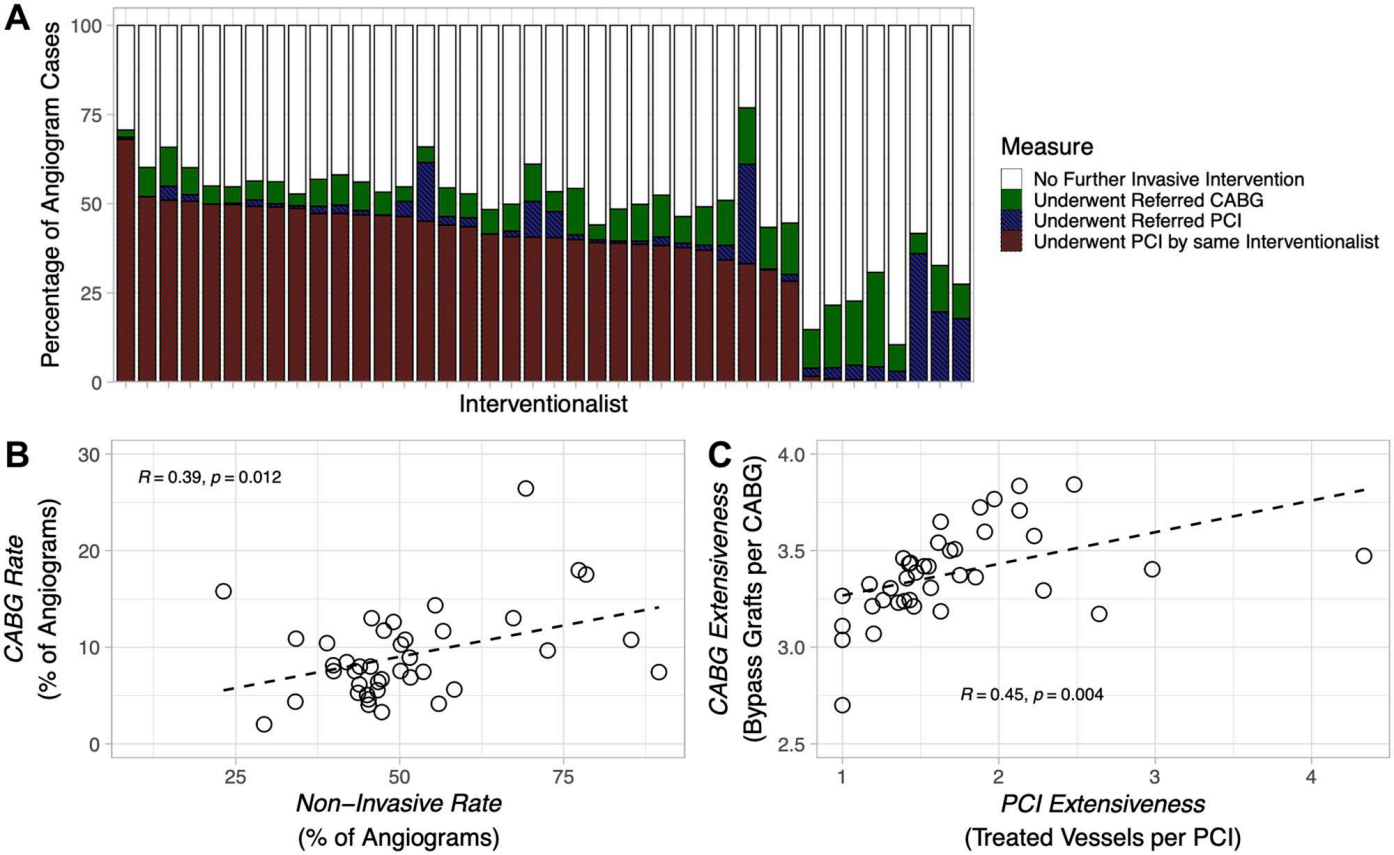
Interventionalist Treatment Extensiveness. (A) Proportion of angiogram cases managed with PCI by the same interventionalist, referred PCI, referred CABG, or no further invasive intervention, with each stacked bar representing one interventionalist. (B) *CABG Rate* versus *Non-Invasive Rate*, with each circle representing one interventionalist. (C) *CABG Extensiveness* versus *PCI Extensiveness*. Abbreviations: CABG, coronary artery bypass grafting; PCI, percutaneous coronary intervention.

**Figure 3A** presents each Interventionalist’s *PCI Rate*, *Referred PCI Rate*, *CABG Rate*, and *Non-Invasive Rate*. Those performing more PCI had fewer patients undergo non-invasive intervention, such that those with higher *Non-Invasive Rates* simultaneously had higher *CABG Rates* (R = +0.39, *P* = .01; **Figure 3B**). *PCI Extensiveness* was correlated with *CABG Extensiveness* (R = 0.45, *P* = .004; **Figure 3C**), meaning those who pursued more extensive PCI referred patients who required a greater number of bypass grafts on average.

### Secondary analysis

**Figure 4.**
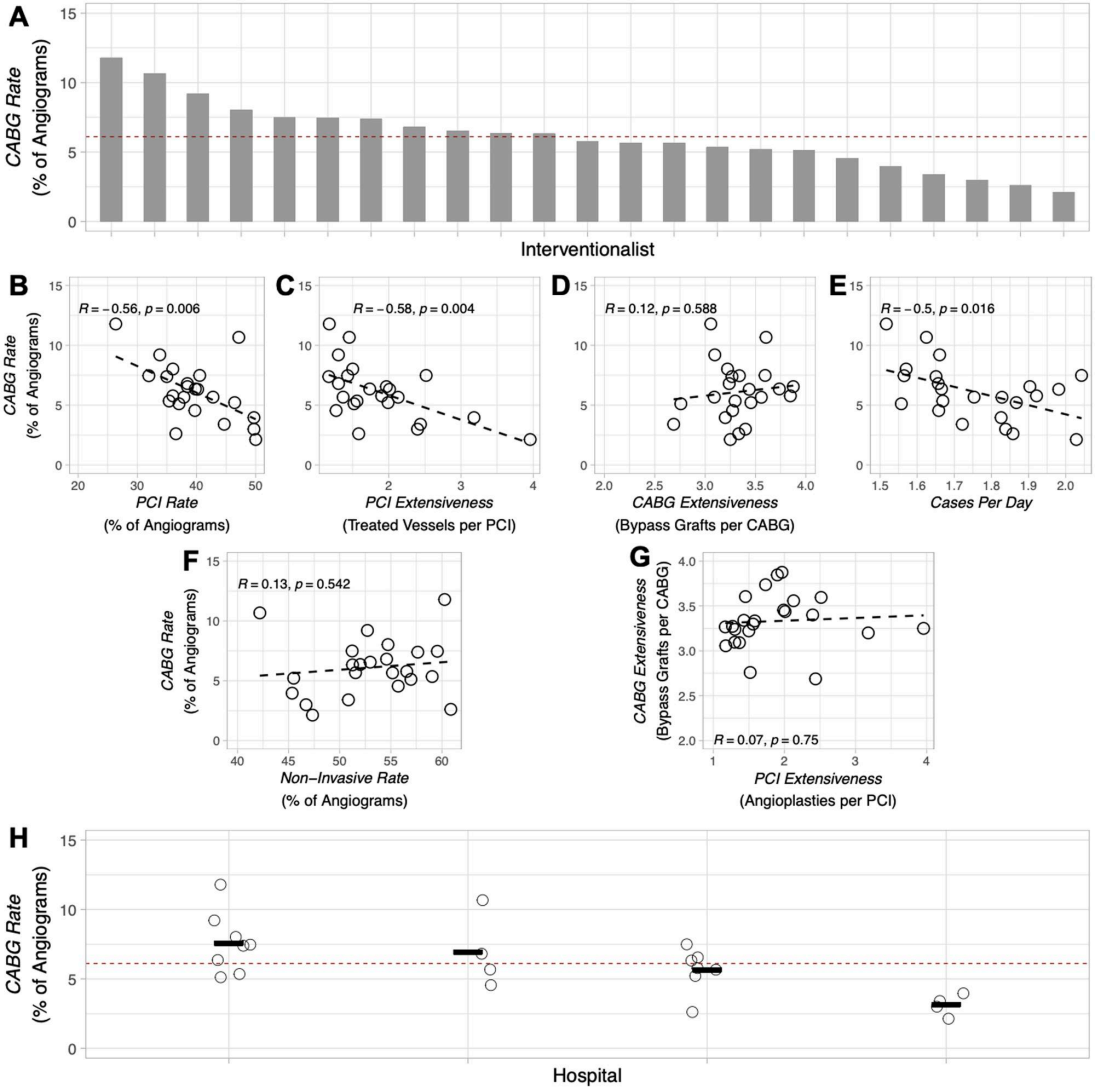
Secondary Analysis in Index MI Patients with Unknown Anatomy. (A) *CABG Rate* by interventionalist. The horizontal dashed reference line indicates the overall mean. (B–F) Associations between *CABG Rate* and other practice parameters, with each circle representing one interventionalist. (G) *CABG Extensiveness* versus *PCI Extensiveness*. (H) *CABG Rate* by hospital. Horizontal summary bars indicate the average CABG Rate for each hospital, and the horizontal dashed reference line indicates the overall study mean. Abbreviations: CABG, coronary artery bypass grafting; PCI, percutaneous coronary intervention.

Within the subgroup of 13 822 angiograms for index acute MI in patients with unknown coronary anatomy, performed by 23 Interventionalists, *CABG Rate* again varied widely across Interventionalists, from 2.1% to 11.8% (mean: 6.66%; **Figure 4A**). Again, the *CABG Rate* was negatively associated with *PCI Rate* (R = −0.56, *P* = .006; **Figure 4B**), *PCI Extensiveness* (R = −0.58, *P* = .004; **Figure 4C**), and *Cases Per Day* (R = −0.50, *P* = .02; **Figure 4E**). However, correlation between *CABG Rate* and both *CABG Extensiveness* (*P* = .59; **Figure 4D**) and *Non-Invasive Rate* (*P* = .54; **Figure 4F**) did not reach significance; *CABG Extensiveness* and *PCI Extensivenes*s were not correlated (*P* = .75; **Figure 4G**). Significant clustering of referred CABG behaviour by centre was again observed (*P* = .02; **Figure 4H**), with ICC of 0.447 (95% CI, 0.028-0.752).

### Clinical outcomes

We identified 73 603 patients for outcome analysis, of which 55 654 (75.6%) underwent PCI on the same date as the initial angiogram, consistent with ad hoc PCI. The remaining 17 949 patients (24.4%) underwent either CABG (15 158; 20.6%) or PCI on a subsequent date (2761; 3.8%). Thus only 24.4% of all revascularizations, and only 4.7% of all PCI cases, could have undergone Heart Team review.

**Figure 5.**
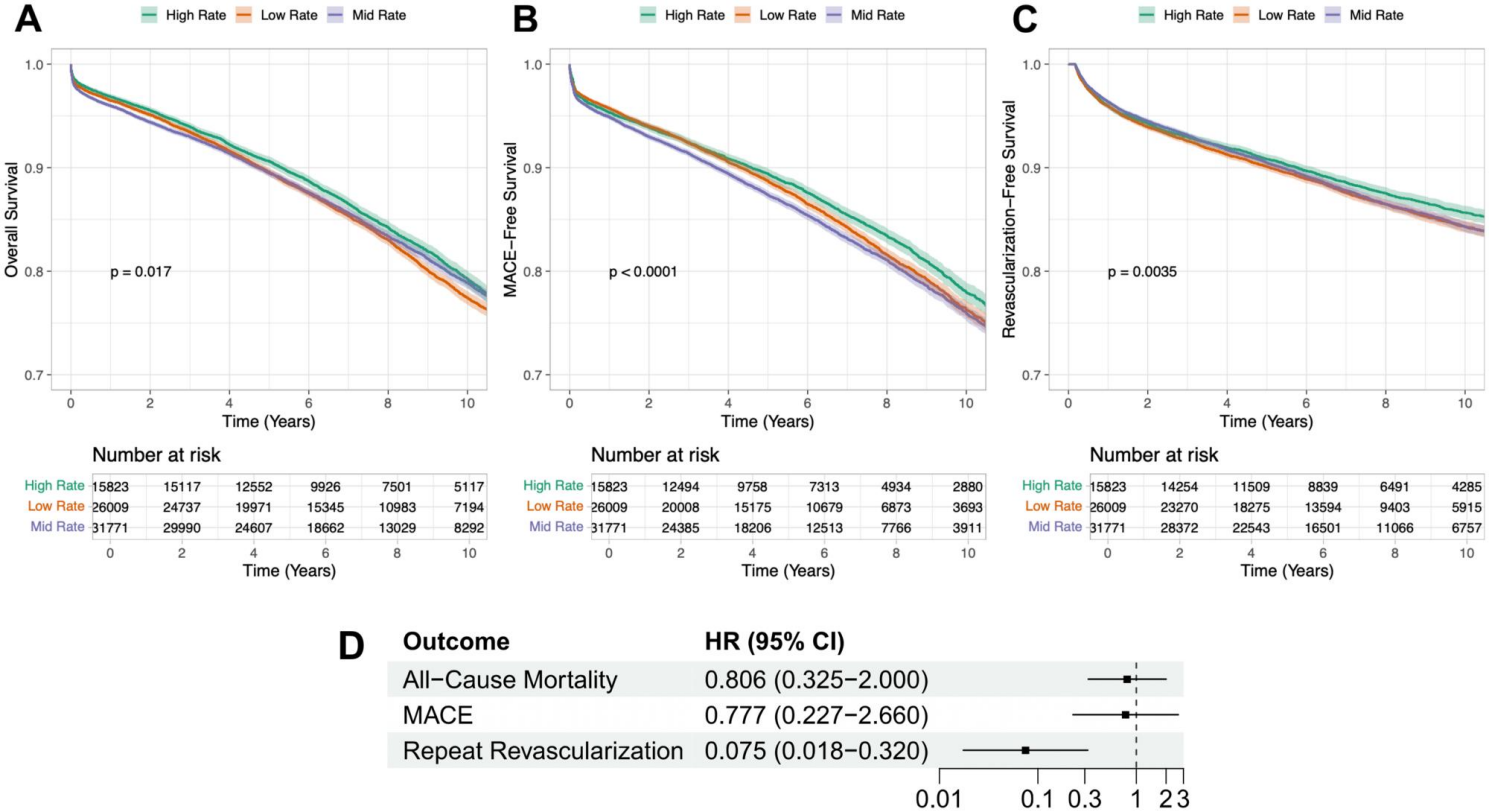
Time-To-Event Survival Analysis. Crude survival curves and numbers at risk for (A) overall survival, (B) MACE, and (C) repeat revascularization, with patients stratified by tertile of angiogram operator *CABG Rate*. (D) Adjusted hazard ratios (HRs) for per unit increase in *CABG Rate* (0 to 100%) across outcomes (log scale). Abbreviations: CI, confidence interval; MACE, major adverse cardiovascular event.

Patients treated by Interventionalists in the highest tertile of *CABG Rate* had significantly lower rates of all-cause mortality (*P* = .02; **Figure 5A**), MACE (*P* < .001; **Figure 5B**), and repeat revascularization (*P* = .004; **Figure 5C**) upon unadjusted analysis. In adjusted Cox regression models (**Figure 5D**), Interventionalist *CABG Rate* was associated with a lower risk of repeat revascularization (hazard ratio [HR] = 0.075, *P* < .001), but was not significantly associated with overall survival (*P* = .64) or MACE-free survival (*P* = .69). Because *CABG Rate* was modelled as a proportional variable (0-1), this HR reflects the effect of an increase from 0% to 100% *CABG Rate* and should not be interpreted as a typical clinical effect size; within the observed range of *CABG Rates* (2.03%-26.4%), this corresponds to an approximately 1.9-fold difference in hazard between patients treated by Interventionalists at the extremes. Importantly, this represents the association between repeat revascularization and Interventionalist-level CABG utilization patterns, rather than a comparison between PCI and CABG revascularization modalities. In fact, the association between *CABG Rate* and repeat-revascularization-free survival persisted in a subgroup analysis of only those patients that underwent CABG (HR = 0.052, *P* < .001), indicating that this association cannot be explained solely by greater use of surgical revascularization.

## Discussion

This study found marked variation in utilization of CABG across Interventionalists that was significantly associated with their frequency and extensiveness of PCI. The negative association between *PCI Extensiveness* and *CABG Rate* may indicate variable adoption of multivessel PCI. The negative association between *Cases Per Day* and *CABG Rate* was unexpected as angiogram with PCI takes longer than angiogram alone; this may reflect more deliberative practices involving more time spent considering management rather than performing PCI.

To our knowledge, no previous study has directly examined the association between Interventionalist PCI practice characteristics and CABG revascularization rates or between Interventionalist-level CABG revascularization rates and clinical outcomes. This study contributes to the small body of evidence of per-Interventionalist analysis across multiple sites using hierarchical statistics to apportion observed variability to hospitals versus the individual Interventionalists.^12^

Variation in the use of CABG versus PCI between hospitals has been previously reported, though our study is much larger and covers a longer time period than prior analyses. Studies from Ontario have demonstrated differences in CABG utilization between centres and have linked higher ad hoc PCI use to worse outcomes.^13^^,^^14^ Variation between cardiologists within a single institution has also been reported.^15^ A prior Alberta-based analysis concluded most variation in revascularization could be explained by patient characteristics^12^; one concern with correcting for such patient factors is that lesions are unlikely to be recorded as insignificant by the same Interventionalist who has gone on to treat them with PCI.

After adjustment for case mix, being treated by an interventional cardiologist with a higher *CABG Rate* was associated with more durable revascularization without differences in survival or MACE, consistent with a prior hospital-level analysis.^12^ We suspect that this reflects better overall matching of patients to the most appropriate revascularization strategy. The persistence of this association within the CABG-only subgroup supports this interpretation: the finding is not simply that CABG patients have lower overall rates of repeat revascularization, but rather that physicians who refer only the most severely diseased patients for CABG may have worse outcomes than physicians who refer a broader, more appropriately selected group of patients for surgery. The absence of a statistically significant association between CABG Rate and mortality or MACE in our analysis should not be interpreted as evidence of equivalence between revascularization strategies, nor does this study represent a direct comparison of CABG versus PCI at the patient level. CABG Rate is an operator-level exposure, and the wide resulting CIs indicate that a clinically meaningful survival benefit cannot be excluded. Accordingly, the findings should not be interpreted as contradicting established randomized trial evidence demonstrating survival advantages of CABG in selected populations,^16^^,^^17^ which are not distinguished within this analysis.

Synthesis of the observed practice patterns supports a broader interpretation beyond comparison of revascularization modalities. Interventionalists with the lowest CABG utilization were characterized not only by lower use of surgery, but also by greater PCI Extensiveness and lower rates of medical management, suggesting a less selective overall approach to revascularization. When considered alongside the higher repeat revascularization rates observed in this cohort without any demonstrable improvement in survival or MACE, this pattern raises the possibility of an “intervention cascade,” in which patients treated by physicians with a lower threshold for extensive immediate PCI are more likely to undergo subsequent revascularization procedures.

Higher *CABG Rate,* therefore, may be best understood as a part of a physician’s practice-pattern “fingerprint”—one aspect of a more restrained and selective approach to revascularization. We have also shown in this study that other characteristics of this fingerprint include a lower rate of proceeding to immediate PCI, less extensive PCI when it is performed, and performing fewer angiographic procedures per day. This fingerprint carries important implications for patient morbidity, healthcare resource use, and the value of care delivered. For cardiac surgeons advocating within Heart Teams, these findings raise the possibility that institutional and per-physician revascularization patterns may represent pragmatic metrics of decision-making selectivity and consistency.

Between-provider variability may reflect differences in Heart Team utilization. Prior evidence demonstrates underutilization of CABG when decisions are made solely by Interventionalists,^18^ even in patients with guideline-directed indications,^19^^,^^20^ particularly in patients with complex disease.^21^ Guidelines emphasize that complex and hybrid revascularization decisions should be based on multidisciplinary Heart Team discussion.^22^^,^^23^ The Team should include an Interventionalist, a general cardiologist, and a cardiac surgeon, supported by regular meetings, structured decision-making, formal documentation, and established communication mechanisms.^24^ Expedited decisions for patients with complex CAD should only be made when delay would compromise care.^24^ Despite broad support of Heart Teams in clinical guidelines, evidence suggests inconsistent implementation.^25^

### Limitations

Our analysis did not account for patient-level clinical or anatomical characteristics such as SYNTAX scores which may explain some of the differences in referral patterns. The number of vessels treated during PCI in administrative billing data does not necessarily correspond to the anatomical extent of coronary artery disease, as billing definitions include branch vessels or multiple segments of the same artery. As such, we cannot determine which revascularization modality choices reflected appropriate clinical decision-making or potential under-referral. However, approaches incorporating lesion severity remain vulnerable to bias, as angiographic stenosis is graded by the treating Interventionalist. Although the secondary analysis restricted the cohort to patients with acute MI, this cohort included both ST-elevation MI and non-ST-elevation MI patients, such that residual variation in case mix between providers may have persisted.

The datasets used in this study do not capture direct referral events between providers, interdisciplinary discussions, surgical wait times, or other system-level constraints that may have influenced *CABG Rate*. We did not directly capture multidisciplinary decision-making processes or the context of clinical discussions, and the possibility of Heart Team involvement was instead inferred using the timing of PCI, an administrative proxy. Our findings are associative and do not permit causal inference regarding underlying determinants. This study relates to a single-payer fee-for-service structure in which physicians are compensated for each additional vessel treated by PCI.^26^

## Conclusion

The likelihood of proceeding to CABG following coronary angiography is strongly influenced by the treating interventional cardiologist, with a 13-fold variation that is associated with other operator practice patterns and cannot be explained by institutional factors alone. Greater use of CABG is associated with more durable revascularization without worse survival or MACE. Those that performed PCI on a higher proportion of cases and treated more vessels in their PCI cases referred fewer patients for CABG. Physician practice “fingerprints,” reflected by consistent patterns of CABG use, PCI selectivity, and procedural intensity, may represent measurable practice-level determinants of downstream patient and healthcare system outcomes. These differences may also reflect variation in the adoption of multivessel PCI and in decision-making approaches, including the extent to which Heart Team processes are incorporated. Over three-quarters of revascularization cases occurred without the possibility of formal Heart Team input. Greater standardization and adherence to structured Heart Team processes may improve consistency of care.

## Supplementary Material

ezag188_Supplementary_Data

## Data Availability

The data that support the findings of this study are held by the British Columbia Ministry of Health and were accessed through Health Data Platform BC under data access approval (24-063). Due to data sharing agreements and privacy restrictions, the underlying row-level data are not publicly available. Access may be granted to qualified researchers through application to Health Data Platform BC, subject to approval by the relevant data stewards and ethics review.

## Abbreviations

CABG: coronary artery bypass graft
CI: confidence interval
ICC: intraclass correlation coefficient
MI: myocardial infarction
PCI: percutaneous coronary intervention
