# Supplementary material for: Association Between Interventional Cardiologist Practice Characteristics, Coronary Artery Bypass Grafting Use, and Clinical Outcomes
Source: Eur J Cardiothorac Surg. 2026 Jun 23;68(7):ezag188. doi: 10.1093/ejcts/ezag188 (PMC13348708; doi:10.1093/ejcts/ezag188)
Supplement: ezag188_Supplementary_Data [file ezag188_supplementary_data.docx]

**Supplement 1**

eAppendix 1: Relevant Fee-For-Service Billing Codes in the Medical Services Plan Billing
 Database

eAppendix 2: Relationship between PCI Billing and Procedural Records

eAppendix 3: Data Subset for Secondary Analysis

**eAppendix 1: Relevant Fee-For-Service Billing Codes** [[24]](https://www.zotero.org/google-docs/?0aQjnh) **in the Medical Services Plan Database**

| **Provincially Insured Service** | **Billing Fee Codes** |
| --- | --- |
| Invasive Coronary Angiogram | S00841, S33131, S33132, S33133* |
| Angioplasty (PCI) | S00840, S00842, S33133*, S33134 |
| Coronary Artery Bypass Graft (CABG) | 07908, 07909 |

***Note:** S33133 is a billing code for combined angiogram and angioplasty.

We restricted the analysis to adults aged 20 to 79 based on the 5-year bin ranges of ages within MSP codes and to focus on adult patients in whom both PCI and CABG are routinely considered viable revascularization strategies; treatment selection in patients aged 80 years and older often differs substantially due to increased operative risk and frailty, which could introduce systematic bias when comparing CABG referral patterns between operators.

**eAppendix 2: Relationship between PCI Billing and Procedural Records**

Within the study jurisdiction, PCI is billed as an integer number of “angioplasty” units on a universal fee-for-service model [[24]](https://www.zotero.org/google-docs/?mZpJL9). Notably, only one “angioplasty” can be billed per unique named vessel treated per case, and the terms are effectively synonymous. For example, multiple short overlapping stents to cover a single lesion would be billed as a single “angioplasty” unit, and would signify a single treated vessel.

In addition to the provincial records detailing these billings, procedural records were available from the provincial clinical program and data administrators, Cardiac Services BC, for all-indication PCI cases taking place during a subset of the study years (January 1, 2013 through December 31, 2020). These records detailed 57,834 unique PCI cases.

Monte Carlo boot-strapped simulation with replacement sampling and 10,000 iterations for the calculation of confidence intervals demonstrated averages of 0.972 stents (95% CI: 0.968-0.977), and 19.1 mm total stent length, per billed “angioplasty” included in fee-for-service billed records (95% CI: 19.0-19.2).

The unique named vessels for which an “angioplasty” can be billed are as follows:

Right Coronary System

- Right coronary artery (RCA)
- Right posterior descending artery (RPDA)
- Right posterior atrioventricular artery
- First right posterolateral artery
- Second right posterolateral artery
- Acute marginal artery
- Inferior septal artery

Left Coronary System

- Left main coronary artery (LM)
- Left anterior descending artery (LAD)
- First diagonal artery (D1)
- Second diagonal artery (D2)
- Ramus artery
- Circumflex artery (LCx)
- First obtuse marginal artery (OM1)
- Second obtuse marginal artery (OM2)
- Third obtuse marginal artery (OM3)
- Left atrioventricular artery
- First left posterolateral artery
- Second left posterolateral artery
- Left posterior descending artery (LPDA)
- First septal artery (S1)

**eAppendix 3: Data Subset for Secondary Analysis**

In order to produce the data subset for secondary analysis, we excluded all patients with prior myocardial infarction (MI) and considered only incident MIs during the study period.

We then excluded any patients who underwent any angiography, percutaneous coronary intervention (PCI), or coronary artery bypass grafting (CABG) prior to their incident MI.

Of the remaining patients, we included only those who underwent invasive angiography (Angiogram) within 5 days of the recorded acute incident MI event.

Finally, we excluded those patients treated by Interventionalists who treated fewer than 50 of these ruled-in patients with PCI.

For each of the included patients, we only counted CABG or PCI procedures occurring within 60 days of the recorded acute incident MI event
